# Cost-Effectiveness of Collaborative Care for the Treatment of Depressive Disorders in Primary Care: A Systematic Review

**DOI:** 10.1371/journal.pone.0123078

**Published:** 2015-05-19

**Authors:** Thomas Grochtdreis, Christian Brettschneider, Annemarie Wegener, Birgit Watzke, Steffi Riedel-Heller, Martin Härter, Hans-Helmut König

**Affiliations:** 1 Department of Health Economics and Health Services Research, Hamburg Center for Health Economics, University Medical Center Hamburg-Eppendorf, Hamburg, Germany; 2 Clinical Psychology and Psychotherapy Research, Institute of Psychology, University of Zurich, Zurich, Switzerland; 3 Institute of Social Medicine, Occupational Health and Public Health, University of Leipzig, Leipzig, Germany; 4 Department of Medical Psychology, University Medical Center Hamburg-Eppendorf, Hamburg, Germany; McGill University, CANADA

## Abstract

**Background:**

For the treatment of depressive disorders, the framework of collaborative care has been recommended, which showed improved outcomes in the primary care sector. Yet, an earlier literature review did not find sufficient evidence to draw robust conclusions on the cost-effectiveness of collaborative care.

**Purpose:**

To systematically review studies on the cost-effectiveness of collaborative care, compared with usual care for the treatment of patients with depressive disorders in primary care.

**Methods:**

A systematic literature search in major databases was conducted. Risk of bias was assessed using the Cochrane Collaboration’s tool. Methodological quality of the articles was assessed using the Consensus on Health Economic Criteria (CHEC) list. To ensure comparability across studies, cost data were inflated to the year 2012 using country-specific gross domestic product inflation rates, and were adjusted to international dollars using purchasing power parities (PPP).

**Results:**

In total, 19 cost-effectiveness analyses were reviewed. The included studies had sample sizes between n = 65 to n = 1,801, and time horizons between six to 24 months. Between 42% and 89% of the CHEC quality criteria were fulfilled, and in only one study no risk of bias was identified. A societal perspective was used by five studies. Incremental costs per depression-free day ranged from dominance to US$PPP 64.89, and incremental costs per QALY from dominance to US$PPP 874,562.

**Conclusion:**

Despite our review improved the comparability of study results, cost-effectiveness of collaborative care compared with usual care for the treatment of patients with depressive disorders in primary care is ambiguous depending on willingness to pay. A still considerable uncertainty, due to inconsistent methodological quality and results among included studies, suggests further cost-effectiveness analyses using QALYs as effect measures and a time horizon of at least 1 year.

## Introduction

In 2010, major depressive disorder (MDD) accounted for 2.5% of the world’s total global burden of disease expressed in disability-adjusted life years (DALY) and ranked second with respect to years lived with disabilities (YLD) [1]. In Europe, lifetime prevalence estimations of MDD range from 11.6% to 17.1% with comorbidities being highly prevalent [2–5].

Mean annual costs per patient with MDD in Europe have been estimated at €3,034, of which €1,251 were due to (non-)medical treatment (direct costs) and €1,782 were due to reduced productivity (indirect costs) [6]. A review of cost-of-illness studies of depression estimated the average annual direct excess costs for a depressed individual at US$1,000 to US $2,500 [7].

MDD is associated with one or more episodes of depressed mood or loss of interest in pleasure in nearly all activities over a period of at least two weeks [8]. MDD requires treatment because otherwise substantial psychosocial problems may occur [9]. Patients with sub-threshold depressive symptoms or mild depression are advised by clinical practice guidelines to be treated with low-intensity psychological interventions and group cognitive behavioral therapy. Patients with moderate to severe depression are advised to be treated either with an antidepressant medication or high-intensity psychological interventions alone, or with a combination of both [10, 11]. The clinical practice guideline of the English National Institute for Health and Clinical Excellence (NICE) [10] also advises to use the framework of a stepped-care model to organize the provision of services, and support patients, carers and physicians in identifying and accessing the most effective interventions. The steps of such a model should consist of psychoeducation, active monitoring, medication and psychosocial interventions.

One way to use the framework of stepped-care and to coordinate care is represented by the collaborative care approach, which is particularly recommended for patients with persistent sub-threshold depressive symptoms or mild to moderate depression with inadequate response to initial interventions, and moderate to severe depression [10]. Collaborative care is a multifaceted intervention that targets patient, physician and structural aspects of care. Treating physicians should be able to coordinate care, guide treatment based on relevant information and synchronize decisions and treatments by ongoing contact with other professionals [12]. Collaborative care was initially developed to improve treatment of depression and short-term clinical outcomes [13]. According to Barkil-Oteo [14], collaborative care improves care for depression in different settings and populations, especially in the primary care sector, which plays a central role in the mental health system and the treatment of depression. Collaborative care for patients with depression was found to be effective in terms of depression outcomes, antidepressant use and quality of life [15–17].

In order to compare the costs and outcomes of collaborative care with usual care or an alternative intervention, cost-effectiveness analyses (CEAs) are applied. In CEAs, a ratio between the differences in costs and the differences in effects of alternative treatments is calculated. One earlier literature review published by van Steenbergen-Weijenburg et al. [18] in 2009 systematically examined cost-effectiveness studies of (stepped) collaborative care for patients with major depressive disorders in the primary care setting. The economic evidence was not sufficient to draw robust conclusions on the cost-effectiveness of collaborative care for patients with depressive disorders. To our knowledge, no more recent systematic review on this topic exists, although several new cost-effectiveness trials on collaborative care for patients with depressive disorders have been published in the last years, such as from the PROMODE study [19], the MDDP study [20] or the TEAM study [21, 22].

The aim of this paper is to systematically review studies on the cost-effectiveness of (stepped) collaborative care compared with usual care for the treatment of patients with depressive disorders in primary care. It provides an update and extension of the literature review by van Steenbergen-Weijenburg et al. [18] by adding recently published studies to the quantitative analysis, improving the comparability of studies by means of inflating and adjusting costs to international dollars, and assessing the quality and risk of bias of included studies.

## Materials and Methods

### Search methods

A systematic literature search was conducted in PubMed, PsychINFO, Embase, Cinahl, EconLit, the Cochrane Library and NHS EED in March 2014 and was updated in February 2015 using a validated rapid review method to minimize time lag of this review [23]. The following search term was used: (depressive disorder OR depression) AND (collaborative care OR disease management OR stepped care) AND (cost-benefit analysis OR cost-effectiveness OR cost-utility OR economic evaluation). Subject headings were additionally used, when applicable. Furthermore, references of studies included in qualitative synthesis and of reviews excluded during eligibility assessment were screened for further eligible studies. The literature search was not limited to any publication year. The studies from the previous review [18] were incorporated in the current review. Articles without abstracts were not included in the data analysis.

### Inclusion of studies

Title and abstract of all articles were independently screened for relevance by two authors (TG and AW). Articles that were deemed relevant were considered in full text. On disagreement, consensus was reached by involving a third author (CB). Full texts of all potentially relevant studies were assessed and included, when

- a cost-effectiveness analysis was presented
- the intervention was (stepped) collaborative care, and
- the study population consisted of patients with depressive disorders.

Articles were excluded if they were protocols, letters, editorials, conference abstracts, case reports, reviews, if the study objectives were other than evaluation of cost-effectiveness of collaborative care, if studies only described decision-analytic models, or if the full text was not available in English or German.

Comorbidity of a depressive disorder and other diseases was accepted if the focus of collaborative care was on depressive disorder. The intervention of the studies had to comply with the definition of (stepped) collaborative care for the treatment of a depressive disorder in primary care provided by van Steenbergen-Weijenburg et al. [18] which closely corresponds to another widely used definition of system level depression management interventions in primary care [24]. Accordingly, programs were defined as collaborative care if treatment complied with at least three of the following four criteria [18]:

1. “Within [(stepped)] collaborative care the role of care manager is introduced to assist and manage the patient by providing structured and systematic interventions.
2. A network is formed around the patient with at least two (…) professionals [(e.g. primary care physician, care manager, and/or consultant psychiatrist)] (…) [13, 25].
3. Process and outcome of treatment is being monitored and in case of insufficient improvement, treatment may be changed according to the principles of stepped care [26].
4. Evidence-based treatment is provided [(e.g. on the basis of a clinical practice guideline [10, 11])] [26].”

### Quality assessment and data abstraction

The risk of bias in studies included in this review was assessed using the Cochrane Collaboration’s tool [27] addressing seven specific domains (sequence generation, allocation concealment, blinding of participants and personnel, blinding of outcome assessment, incomplete outcome data, selective outcome reporting and ‘other issues’). For the economic evaluation aspects of the articles, the Consensus on Health Economic Criteria (CHEC) list for economic evaluations [28] was used as a quality criteria list. The CHEC-list addresses 19 categories assessing methodological quality of economic evaluations [28]. If necessary, information was retrieved from related studies or protocols when they were stated as source. Two authors (TG and AW) independently assessed the risk of bias of the studies as well as the methodological quality of the economic evaluations. Discussion or third opinion (CB) was used in case of disagreement. Independently from the methodological quality and the risk of bias of each study, all available evidence was used for analysis to avoid loss of information. Risk of bias data was processed graphically with Review Manager 5.3 [29].

All abstracted data (e.g. perspectives, effect measurements, cost measurements, incremental cost-effectiveness or cost-utility ratios) from each selected study was entered into spreadsheets.

### Analysis of included studies

As summary measures, incremental cost-effectiveness ratios (ICERs) in terms of incremental costs per depression-free day (DFD), per quality-adjusted life years (QALYs) or per other outcomes were reported.

To ensure comparability across studies, cost data were inflated to the year 2012, using country-specific gross domestic product (GDP) inflation rates [30], and were adjusted to international dollars using GDP purchasing power parities (US$PPP) [30]. If no reference year for cost valuation was given, the middle of the follow-up period was used as reference for inflation. If the follow-up period was not reported, the publication year was used as reference. Cost measurements were classified into two different perspectives of economic evaluations according to the Consolidated Health Economic Evaluation Reporting Standards (CHEERS) [31]: the health-care system perspective or the societal perspective, respectively. Incremental cost-effectiveness ratios were not pooled since there are no accepted methods of pooling [32].

## Results

### Study selection

**Fig 1:**
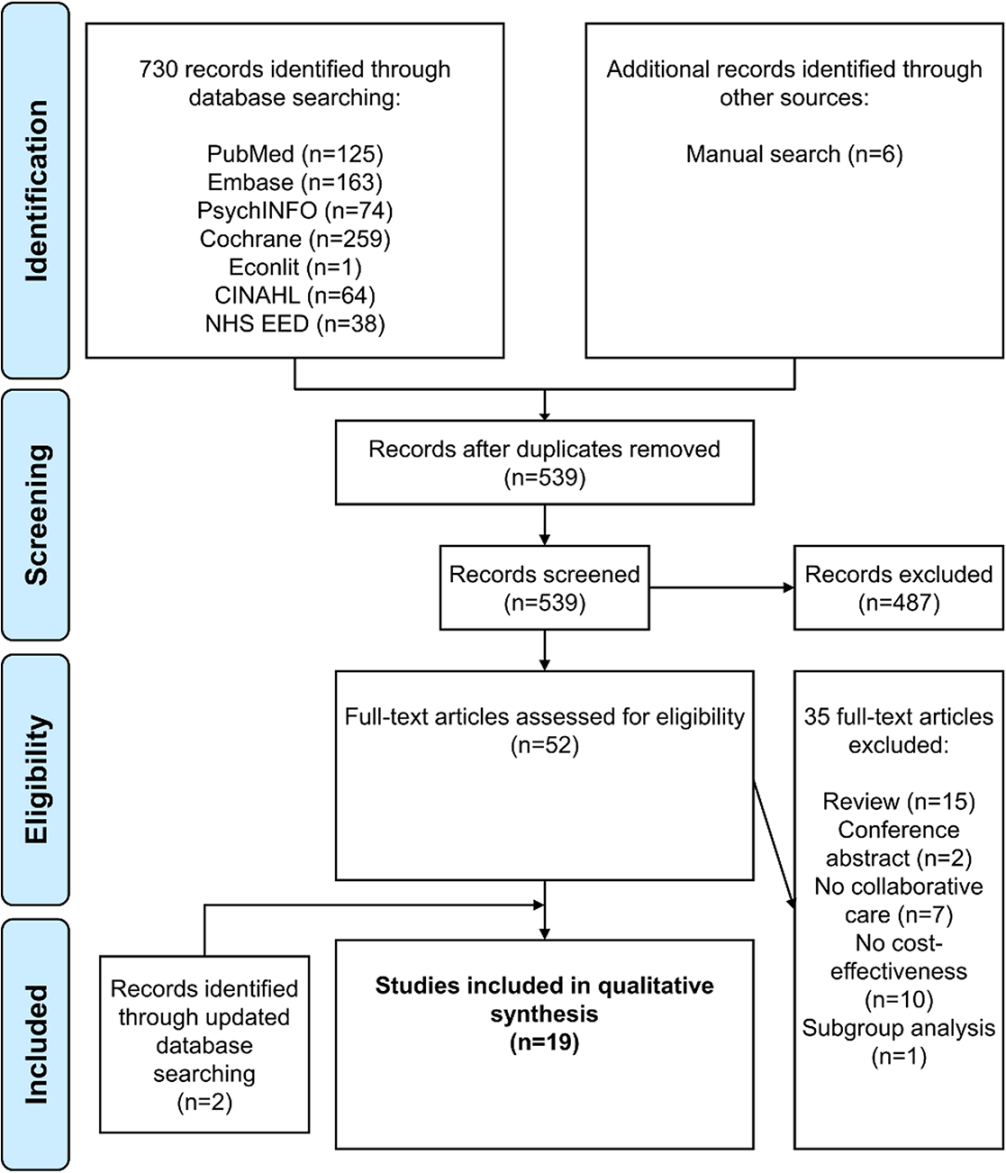
Flow chart of the selection process based on the PRISMA Statement [75].

In total, 736 articles were identified. Based on title and abstract screening for relevance, 197 duplicates and 487 non-relevant articles were removed. From the remaining 52 potentially relevant articles, full texts were retrieved and examined for relevance. Thirty-five were rejected (15 reviews, 2 conference abstracts, 7 no collaborative care, 10 no cost-effectiveness analysis, 1 only subgroup analysis). An update of the systematic literature search identified two additional articles. Finally, 19 studies were included in the review [33–51]. Through the search, all eight studies included in the literature review by van Steenbergen-Weijenburg et al. [18] were identified. A flow chart of the selection process is presented in Fig 1.

### Study characteristics

The general characteristics of the included CEA are presented in Table 1. The included studies originated from the United States (n = 12), the Netherlands (n = 4), Chile (n = 1), Spain (n = 1) and the United Kingdom (n = 1). The earliest study was published in 1998 [49] and the most recent were published in 2014 [33, 50, 51]. All but three studies were multicenter trials conducted in primary care clinics (n = 10), primary care practices (n = 7), residential homes (n = 1) or an occupational health care setting (n = 1). The mean number of centers was 22, ranging from one to 89 centers.

**Table 1:** General characteristics of the included studies.

Study	Population	Sample size (IG/CG)	Mean age IG/CG (SD)	% Female IG/CG	Setting (n)	Country
**Aragonès et al. 2014 [33, 76, 77]**	Patients with major depression	338 (166/126)	47.5 (15.5) / 47.8 (14.9)	81.0 / 77.2	PCC (20)	Spain
**Araya et al. 2006 [34]**	Female patients aged 18–70 with major depression	240 (96/95)	44.1 (12.1) / 42.0 (13.7)	100.0	PCC (3)	Chile
**Bosmans et al. 2014 [35]**	Elderly residential home residents at risk of major depression and/or anxiety disorder	185 (93/92)	84.0 (6.7) / 84.0 (6.4)	72.0 / 74.0	Residential homes	NL
**Donohue et al. 2014 [51, 78, 79]**	Post-CABG patients with depressive symptoms	189 (90/99)	66.9 (9.0) / 67.1 (11.5)	36.0 / 41.0	PCP	USA
**Goorden et al. 2013 [36, 80]**	Sick-listed employees with major depression	126 (65/61)	41.9 (11.4) / 43.4 (11.4)	53.8 / 54.1	Occupational Health Care	NL
**Green et al. 2014 [50, 81, 82]**	Patients with major depression	581 (276/305)	45.0 (13.2) / 44.5 (13.4)	73.2 / 70.8	PCP (49)	UK
**Hay et al. 2012 [20, 37]**	Low income patients with major depression and diabetes	387 (193/194)	*Not given*	85.5 / 79.8	PCC (2)	USA
**Katon et al. 2012 [38, 83]**	Patients with major depression and diabetes and/or CHD	214 (106/108)	57.4 (10.5) / 56.3 (12.1)	48.0 / 56.0	PCC (14)	USA
**Katon et al. 2005 [39, 84, 85]**	Patients aged ≥60 with major depression and/or dysthymia	1801 (906/895)	71.0 (7.4) / 71.4 (7.5)	65.0 / 64.0	PCC (18)	USA
**Liu et al. 2003 [40]**	Patients with major depression and/or dysthymia	354 (168/186)	57.8 (13.5) / 56.6 (14.2)	95.0 / 96.0	PCC (1)	USA
**Pyne et al. 2010 [21, 22, 41]**	Patients with major depression	320 (141/179)	58.8 (11.4) / 60.0 (11.7)	5.0 / 11.0	PCP (89)	USA
**Rost et al. 2005 [42, 86, 87]**	Patients with major depression	211 (115/96)	43.1 (14.8)	84.4	PCP (12)	USA
**Schoenbaum et al. 2001 [43, 88, 89]**	Patients with major depression	1356 (424+489/ 443)	44.5 (15.5) / 42.2 (13.9)	71.6 / 69.0	PCP (48)	USA
**Simon et al. 2007 [44, 90, 91]**	Patients with major depression and diabetes	329 (165/164)	58.0 (12.0) / 57.0 (12.0)	35.0 / 34.0	PCC (9)	USA
**Simon, Katon et al. 2001 [45, 92]**	Patients with depressive symptoms	228 (110/109)	47.0 (14.0)	74.0	PCC (4)	USA
**Simon, Manning et al. 2001 [46]**	Patients with major depression	407 (218/189)	45.6 (8.6) / 45.4 (9.6)	77.0 / 78.0	PCC (7)	USA
**van der Weele et al. 2012 [47]**	Patients aged ≥75 with untreated depressive symptoms	239 (121/118)	80.0 / 80.0	70.0 / 75.0	PCP (67)	NL
**van't Veer-Tazelaar et al. 2010 [48, 93]**	Patients aged ≥75 at risk of major depression and anxiety disorder	170 (86/84)	81.8 (3.8) / 81.1 (3.5)	69.8 / 77.4	PCP (33)	NL
**Von Korff et al. 1998 [13, 49, 52]** *	Patients with major depression	91 (49/42)	43.2 (15.4) / 42.3 (12.7)	77.5 / 88.1	PCC (1)	USA
		65 (31/34)	43.1 (9.3) / 44.8 (15.9)	77.4 / 73.5		

CABG = Coronary Artery Bypass Graft, CHD = Coronary Heart Disease, IG = Intervention Group, CG = Control Group, PCC = Primary Care Clinic, PCP = Primary Care Practice, NL = the Netherlands, UK = United Kingdom

*Analysis was based on two RCT

The sample size varied from N = 65 in one trial with a single center to N = 1,801 in a trial with 18 centers (mean sample size N = 392). All studies focused on patients with depressive disorders alone, with depressive symptoms or at risk of depressive disorders. Three studies only included patients with comorbid diabetes [37, 44], or both, diabetes and coronary heart disease [38]. One study only included patients with depressive symptoms following coronary artery bypass graft [51]. The mean age of the patients varied from 42 years to 84 years (overall mean age 56 years). Four studies only included aged patients with an overall mean age of 79 years [35, 39, 47, 48]. The overall mean percentage of included female patients was 68%, ranging from 8% in the study by Pyne et al. [41], where the setting was a veteran population, to 100% in the study by Araya et al. [34], where only women were included on purpose.

A societal perspective was used by five studies [35, 36, 43, 47, 48] and eleven studies used a health care perspective [34, 36–41, 44, 45, 49–51]. Both, a health care perspective and a societal perspective, was used by three studies [33, 42, 46].

All studies indicated that a care manager assisted and managed the patient by providing structured and systematic interventions. In ten studies, the care management was implemented by nurses/health care professionals solely [33, 38, 40–45, 51] or, in addition, social workers [34] or psychologists [39, 50], respectively. Physicians were care managers in three studies [35–37].

In all studies, a network was formed around the patient comprising at least two professionals [34–36, 43, 45, 46, 49]. In ten studies, the network was composed of three professionals [33, 37–40, 42, 47, 48, 50, 51] and in two studies, the network was composed of four to five different professionals [41, 44]. Professionals routinely associated with the network were mainly primary care physicians [33, 35–47, 49–51], specialized nurses [33–35, 38, 39, 41–44, 47, 48, 51] and psychiatrists [38–40, 44, 45, 49, 51]. Other professionals associated with the network were psychologists [39, 40, 44, 50], social workers [34, 37, 40] and pharmacologists [41].

Monitoring was an element of collaborative care in all but two studies [35, 47]. Treatment progress and response was monitored in nine studies [34, 36, 38–40, 46, 49–51] and medication or treatment adherence in twelve studies [33, 34, 40–46, 49–51]. Symptoms were being monitored in six studies [34, 37, 41–43, 48] and adverse effects were being identified through monitoring in three studies [33, 41, 46]. Treatment of patients was adapted according to the principles of stepped care in eleven studies [34–37, 39–41, 44, 47, 48, 51].

Evidence-based treatment was provided in all studies. The majority of studies provided patients in the collaborative care group with antidepressant pharmacotherapy [33, 34, 36, 37, 39–46, 49–51]. Further evidence-based treatments used in the studies were psychoeducation [33, 34, 37–40, 42, 43, 45, 46, 49, 51], psychotherapy (e.g. cognitive behavior therapy) [35, 36, 40, 42–45, 47–50], counseling [34, 35, 40, 41, 44–47, 49, 51] and problem-solving treatment [36–39, 44, 48, 49].

All but two studies used patients with usual care as control group. One study presented patients in the control group with depression educational pamphlets and community service resource lists additionally to usual care [37]. The control group patients in another study were advised to consult with their primary physician to receive care for depression beyond usual care [38].

Ten studies reported cost adjustment by usage of reference unit prices for a certain year [35–37, 40–43, 48, 50, 51] and three studies stated that there was no need to discount cost data because of a short follow-up [33, 34, 47].

### Quality and risk of bias assessment

Between 42% and 89% of the CHEC-list criteria [28] were fulfilled by the studies. The mean quality criteria fulfillment was 69%. Four studies [33, 35, 36, 47] were able to address almost all methodological quality criteria from the CHEC-list, two studies [45, 49] failed to address the majority of these quality criteria. Results of quality assessment based on the CHEC-list are presented in S1 Table.

No risk of bias was identified in the study by Aragonés et al. [33]. High risk of attrition bias was identified in two studies [35, 39]. Other biases, such as a too small sample size, a high proportion of missing cost data, randomization imbalances or crossover, appeared in nine studies [35–37, 39, 43–45, 49, 51]. Results for the authors' judgments on risk of bias items for each included study and for each risk of bias item as percentages across all included studies are presented in S1 and S2 Figs.

### Effects

#### Depression-free days

More than half of the studies reported incremental DFDs [34, 38–40, 44–46] or, both, DFDs and QALYs [33, 42, 51]. The studies which reported DFDs as their primary effect measure calculated them using the Hamilton Rating Scale for Depression (HDRS) [34, 46, 51], the 20-item Hopkins Symptom Checklist Depression Scale (HSCL-20) [39], the Patient Health Questionnaire (PHQ-9) [33], or the Symptom Checklist-90 (SCL-90) [38, 40, 44, 46]. The study by Rost et al. [42] used directly reported depression impairment-free days. For a follow-up period of less than twelve months, the lowest/highest reported effects were 14.6 [40] and 50 [34] incremental DFDs, respectively. For a follow-up of twelve months (24 months) the lowest/highest reported effects were 20 [51] and 47.4 [46] (48 [44] and 107 [39]) incremental DFDs, respectively. In two studies with DFDs as effect measure, the incremental effect was not statistically significant [40, 51]. The main findings concerning the cost-effectiveness of collaborative care vs. usual care of the included studies are summarized in Table 2.

**Table 2:** Cost-effectiveness of collaborative care vs. usual care.

Study	Follow up in months	Perspective	Classification	Reference year	Incremental Effects^§^ (95% CI)	Converted incremental Costs per US$PPP (95% CI)	Converted ICER in US$PPP (95% CI)
**Effect measure: Depression-free days**							
**Aragonès et al. 2014 [33, 76, 77]**	12	HCP	PHQ-9	2009*	40.1^†^	260.99 (SD 76.01)^‡^	6.51
		SP				225.12 (SD 222.23)	5.62
**Araya et al. 2006 [34]**	6	HCP	HDRS	2004**	50^†^	47.29 (30.78 to 67.68)^†^	0.94 (0.56 to 1.49)
**Katon et al. 2012 [38, 83]**	24	HCP	SCL-90	2009*	114 (79 to 149)^†^	−623.51 (−3,590,95 to 3,020.97)	Dominant (−31.24 to 20.12)
**Donohue et al. 2014 [51, 78, 79]**	12	HCP	HDRS	2004	20 (-8 to 48)	−528.70	Dominant (−75.36 to −36.50)
**Katon et al. 2005 [39, 84, 85]**	24	HCP	HSCL-20	2005***	107 (86 to 128)^†^	788.43 (1,520.95 to 3,097.82)	56.59 (−17.30 to 131.19)
**Liu et al. 2003 [40]**	9	HCP	SCL-90	2000	14.6 (−0.5 to 29.6)	216.66 (−2,373.02 to 3,144.80)	2.56 (−325.63 to 510.24)
**Simon et al. 2007 [44, 90, 91]**	24	HCP	SCL-90	2007***	48 (23 to 73)^†^	−338.63 (−1,086.00 to 408.73)	Dominant (−18.98 to 7.76)
**Simon, Katon et al. 2001 [45, 92]**	6	HCP	SCL-90	2001***	16.7 (1.3 to 31)^†^	731.44 (619.97 to 2,077.85)^†^	37.64 (-64.79 to 485.38)
**Simon, Manning et al. 2001 [46]**	12	HCP	HDRS	2001***	47.4 (26.6 to 68.2)^†^	2,472.38 (1,062.09 to 3,144.80)^†^	51.78 (20.09 to 101.49)
		SP				3,099.86 (1,102.17 to 5,182.72)^†^	64.89 (21.76 to 135.86)
**Effect measure: QALY**							
**Aragonès et al. 2014 [33, 76, 77]**	12	HCP	SF-6D	2009*	0.045 (SD 0.019)^†^	260.99 (SD 76.01)^‡^	5,800
		SP				225.12 (SD 222.23)	5,003
**Bosmans et al. 2014 [35]**	10	SP	EQ-5D	2008	0.03 (−0.03 to 0.09)	1,083.11 (−766.45 to 3,127.83)	34,755
**Goorden et al. 2013 [36, 80]**	12	SP	EQ-5D	2009	−0.05 (−0.11 to 0.00)	−915.51	18,838 (per QALY gained by usual care)
**Donohue et al. 2014 [51, 78, 79]**	12	HCP	SF-6D	2004	0.05 (0.02 to 0.08)^†^	−528.70	Dominant (−14,059 to −9,229)
**Green et al. 2014 [50, 81, 82]**	12	HCP	EQ-5D	2011	0.019 (−0.019 to 0.06)	391.04 (−293.19 to 1,279.82)	20,580
			SF-6D		0.0168 (0.000 to 0.032)		23,276
**Hay et al. 2012 [20, 37]**	18	HCP	SF-12	2009	0.13^‡^	540.58^†^	4,254
**Katon et al. 2012 [38, 83]**	24	HCP	Regression model^¶^	2009*	0.335 (−0.18 to 0.58)	−623.51 (−3,590.95 to 2,154.99)	Dominant (−3,021 to 3,021)
**Pyne et al. 2010 [21, 22, 41]**	12	HCP	EQ-5D	2005	0.018^†^	1,854.66^‡^	153,299
**Rost et al. 2005 [42, 86, 87]**	24	SP	Regression model (based on DFDs)	2000	0.049^†^	898.70 (816.65 to 980.75)^‡^	18,341
**Schoenbaum et al. 2001 [43, 88, 89]**	24	SP	SF-12	1998	0.0115 (−0.004 to 0.027)	557.20 (−621.03 to 1,736.77)	48,495 (QI-meds)
					0.0226 (0.008 to 0.038)^†^	644.97 (−522.63 to 1,736.77)	28,562 (QI-therapy)
					0.0173 (0.004 to 0.030)^†^	603.75 (−405.60 to 1,614.42)	34,899 (pooled)
**van der Weele et al. 2012 [47]**	12	SP	SF-12	2001*	0.008	6,996.50	874,562 (age 75–80)
					0.02	−859.59	Dominant (age ≥80)
			EQ-5D	2001*	−0.021	6,996.50	Dominated (age 75–80)
					0.044	−859.59	Dominant (age ≥80)
**Other effect measures**							
**van't Veer-Tazelaar et al. 2010 [48, 93]**	12	SP	Depression-/anxiety-free year	2007	0.12 (0.01 to 0.24)^†^ probability for a beneficial outcome	702.87	5,677 (−1,175 to 35,774) per depression/anxiety-free year
**Von Korff et al. 1998 [13, 49, 52]** ^**&**^	7	HCP	Successfully treated case (SCL-90)	1995***	30.6% successfully treated cases of major depression	677.64	2,215 per successfully treated case of major depression
				1996***	28.1% successfully treated cases of major depression	497.39	1,284 per successfully treated case of major depression

HCP = Health Care Perspective; SP = Societal Perspective; DFD = Depression-free day; QALY = Quality-adjusted life year; PHQ-9 = Patient Health Questionnaire; HDRS = Hamilton Rating Scale for Depression; SCL-90 = Symptom Checklist-90; HSCL-20 = 20-item Hopkins Symptom Checklist Depression Scale; SF-12 = 12-Item Short Form Health Survey; CLP = Chilean Pesos; ICER = Incremental Cost-Effectiveness Ratio; QI-meds = quality improvement—medical management; QI-therapy = quality improvement—psychotherapy

^&^Analysis was based on two RCT

^§^Based on follow up in months

^¶^QALYs were estimated based on age, sex, microalbuminuria, HbA_1c_, LDL-C and systolic blood pressure levels

*Based on the middle of the follow up period

**Based on the year of article receipt by journal

***Based on the publication year

^†^significant with p<0.05

^‡^significant with p<0.00

#### QALYs

QALYs were reported by more than half of the studies [33, 35–38, 41–43, 47, 51]. Both [47, 50] or either the EQ-5D [33, 36, 41] and the Short Form Health Survey (SF-12/SF-6D) [33, 37, 43, 51] were used to calculate QALYs. Two studies generated QALYs based on age, gender plus clinical measures [38] and DFDs [42], respectively, using a regression model. For a follow-up of ten to twelve months (18 to 24 months), the lowest positive effect was 0.008 [47] (0.0115 [43]) additional QALYs and the highest effect was 0.05 [51] (0.335 [38]). Two studies found incremental QALYs for usual care compared to collaborative care of 0.05 [36] and 0.021 [47], respectively. In four studies with QALYs as effect measure, the incremental effect was not statistically significant [35, 36, 49, 50].

#### Other effects

One study, which examined the preventive effect of stepped collaborative care for people at risk for depression and anxiety disorders, reported a probability of a depression/anxiety-free year of 0.88 in the collaborative care group and of 0.76 in the usual care group, respectively, leading on to an incremental effectiveness of 0.12. [48]. In another study, which examined the improvement in major depression status through collaborative care based on two clinical trials, 30.6% [13] and 28.1% [52], respectively, more patients in the collaborative care group improved compared to patients with usual care [49]. However, no statistical significance testing was reported for incremental effectiveness.

### Costs

#### Direct costs

All but two studies included medication and outpatient care costs. The studies of Green et al. [50] and Donohue et al. [51] excluded medication costs due to difficulties in data collection. Some studies also considered inpatient care costs [33, 35, 37–39, 41, 45, 46, 50, 51] and non-medical and paramedical costs [36, 47, 48, 50]. Of all studies, 76% reported positive incremental direct costs of collaborative care with a range between US$PPP 46 [34] to 3,761 [41]. Negative incremental direct costs were reported with a range between US$PPP −529 [51] to −982 [44] in favor of the collaborative care group. However, twelve studies either reported non-significant incremental costs [35, 38–40, 43, 44, 47, 48, 50, 51] or no statistical significance testing was reported for incremental costs [36, 49], respectively. A summary of direct cost elements and mean costs of the studies is given in S2 Table.

The mean intervention cost of all studies ranged between US$PPP 90 [33] to 1,269 [38], except for the study by Araya et al. [34] which reported intervention costs of only US$PPP 19. The intervention components ranged from only additional individual consultations from care managers [33, 36, 46, 47, 49] to complex (stepped) collaborative care interventions [34, 35, 38, 39, 43, 48, 51]. A detailed description of the intervention costs is given in S3 Table.

#### Indirect costs

Aragonès et al. [33] reported mean indirect costs of temporary disability leave from work amounting to US$PPP 899 (930) in the collaborative care group (usual care group). The mean productivity costs as reported by Goorden et al. [36] were US$PPP 14,920 (17,158). These costs consisted of US$PPP 1,988 (2,441) for absenteeism and US$PPP 13,065 (15,947) for presenteeism, respectively [36]. Three studies interpreted patient time and travel costs as indirect costs [36, 37, 41], and one of these studies only indicated that they ascertained indirect costs for their economic evaluation but did not report them separately [37].

### Cost-effectiveness

#### Cost-effectiveness per depression-free day

**Fig 2:**
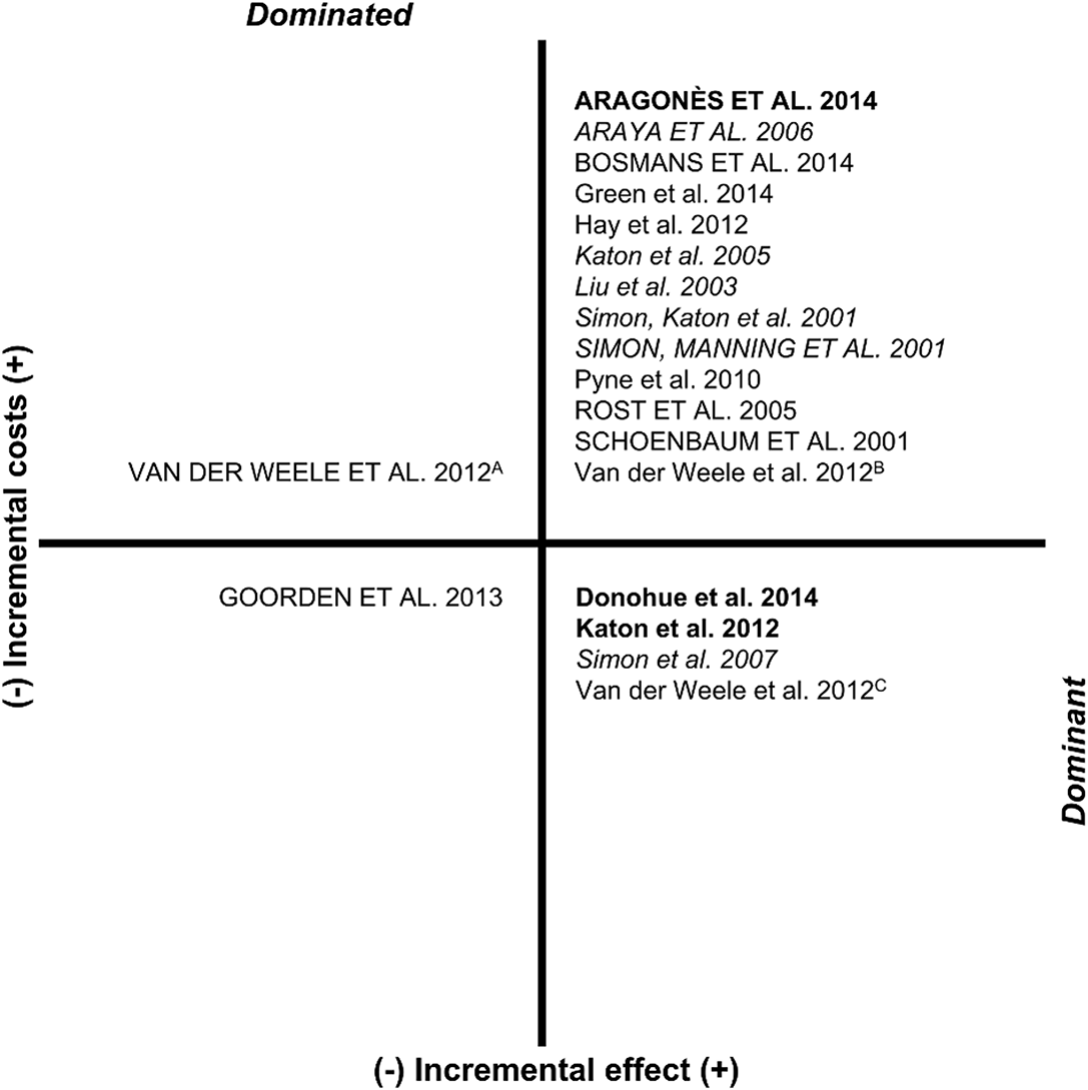
Cost effectiveness plane for studies with costs per DFD/QALY. In capitals: studies with a societal perspective, in italics: studies with costs per DFD, in bold: studies with costs per DFD and QALY, ^A^Patients aged 75–80, effectiveness measurement instrument EQ-5D; ^B^Patients aged 75–80, effectiveness measurement instrument SF-12, ^C^Patients aged ≥80.

All but three studies with DFDs as effect measure reported that collaborative care is more effective in terms of additional DFD, but also more expensive [33, 34, 39, 40, 45, 46]. From a health care perspective, ICERs ranged from dominance [38, 44, 51] to US$PPP 56.59 [39] per additional DFD. From a societal perspective, the ICERs ranged from US$PPP 5.62 [33] to 64.89 [46] per additional DFD. The directions of the differences in costs and depression free days (and QALYs) are summarized in a cost-effectiveness plane (Fig 2).

#### Cost-effectiveness per QALY

The majority of studies with QALYs as effect measure reported that collaborative care is more effective in terms of additional QALYs, but also more expensive [33, 35–37, 41–43, 47, 50] (Fig 2). From a health care perspective, ICERs ranged from dominance [38, 51] to US$PPP 153,299 [41] per additional QALY. From a societal perspective, ICERs ranged from dominance [47] to US$PPP 874,562 [47] per additional QALY. The study by van der Weele et al. [47] reported that collaborative care dominated usual care in patients aged >80 years, but was dominated by usual care in patients aged 75 to 80 years. The study by Goorden et al. [36] found higher costs and higher effects for usual care compared to collaborative care, with an ICER of US$PPP 18,838 per additional QALY.

#### Cost-effectiveness per other outcome

One study of stepped collaborative care for people aged ≥75 at risk for depression and anxiety disorders indicated that collaborative care is more effective in terms of preventing depression/anxiety disorders but also more expensive compared to usual care [48]. The ICER was US$PPP 5,677 per depression/anxiety-free year. Another study indicated that collaborative care for depressed primary care patients is more effective in terms of successfully treated cases but also more expensive compared to usual care [49]. Based on two clinical trials, the ICER was US$PPP 2,215 (1,284) per case successfully treated. Neither of these two studies reported, both, a significant incremental effect and significant incremental costs.

## Discussion

This study reports a systematic review of cost-effectiveness analyses of (stepped) collaborative care compared with usual care for the treatment of patients with depressive disorders in primary care. In 13 of the 19 included studies, collaborative care was associated with better effects and higher costs (Fig 2). Across the studies showing a higher effectiveness in terms of additional QALYs, collaborative care was associated with ICERs ranging from dominance to US$PPP 153,299 from a health care perspective and from dominance to US$PPP 874,562 from a societal perspective. Across the studies showing a higher effectiveness in terms of additional DFD, collaborative care was associated with ICERs ranging from dominance to US$PPP 56.59 from a health care perspective and from US$PPP 5.62 to 64.89 from a societal perspective.

Compared with incremental costs per additional QALY for collaborative care reported in the review by van Steenbergen-Weijenburg et al. [18] (US$ 21,478 to 49,500), the current range is considerably broader. All three studies with dominant ICERs used a health care perspective, had a follow-up period of twelve to 24 months and were conducted in populations of depressive patients with comorbid diseases or patients with post-surgery depression, respectively [38, 44, 51]. Yet, the study by Katon et al. [38] estimated QALYs based on a regression model and the ICER showed a wide confidence interval. The ICER of the study by Pyne et al. [41] (US$PPP 153,299), which exceeded the frequently applied cost-effectiveness threshold of US$ 50,000 per additional QALY [53], resulted from high costs and modest effectiveness of collaborative care. The ICER of the study by van der Weele et al. [47] (US$PPP 874,562 for patients aged 75–80) resulted from a very small and non-significant effectiveness of collaborative care.

Across all studies included in this review, the time horizons of the economic evaluations as well as the inclusion of indirect costs in the cost calculation varied considerably. Studies with time horizons of more than one year had incremental costs per QALY gained ranging from dominance to US$PPP 34,899, studies with time horizons of one year and below had incremental costs per QALY gained ranging from dominance to US$PPP 874,562. There might be a trend showing better ICERs in studies with longer time horizons. Yet, ICERs may be influenced not only by the time horizons but also by the intervention elements, the included cost elements (e.g. inpatient costs, medication costs) and the size of health effects. In fact, the range of health effect sizes in studies with time horizons of more than one year was 0.0173 to 0.335 incremental QALYs, and in studies with time horizons one year and below it was −0.05 to 0.05 incremental QALYs. According to the American Psychiatric Association [12], the average duration of MDD is between 16 to 24 weeks. However, MDD is recurrent in around 40% of patients within two years and unremitting in at least 15%, leading to persistent residual symptoms and social or occupational impairment [12, 54, 55]. Around 5% to 10% of patients have a continuous MDD for 2 or more years, illustrating the high risk of chronification [8]. Therefore, time horizons of at least one to two years would be desirable, despite the high costs of clinical trials with a long follow-up.

Only two included studies [33, 36] reported indirect costs of lost productivity, even though eight studies used a societal perspective. However, none of those studies identified an effect of depression treatment on indirect costs. This may be explained by the limited time horizon of one year and by the inability to include presenteeism and unpaid work in the estimation of indirect costs [33, 36]. Yet, lost productivity has been reported to cause the largest share in total costs of patients with depression [7, 56, 57]. However, there is an ongoing debate on whether to include indirect costs in economic evaluations [58, 59] and various national guidelines for economic evaluations mainly recommend a health care payer’s perspective [60–63].

Methodological quality varied across studies. The range of scores on the 19-item CHEC-list [28] was from eight to 17 points. Notably, the quality of the included studies improved over time. Studies published before 2009 (47%) had a mean score of 12 points and studies published after 2009 (53%) had mean score of 15 points. Seven studies reported neither significant incremental effects nor significant incremental costs, possibly attributable to an insufficient sample size [35, 36, 40, 47, 49–51]. Two studies themselves indicated that their cost-effectiveness analyses were underpowered [35, 49], which is a common problem of economic evaluations conducted alongside clinical trials [64–66]. In order to further improve quality of economic evaluations it is suggested to conduct cost-effectiveness analyses based on samples large enough to be confident in the resulting cost-effectiveness estimates [67], as it is anticipated in the study embedded in the intersectoral research network “psychenet: Hamburg Network for Mental Health (2011–2014)” [68].

The QALY valuation methods used across studies were mainly based on utility scales such as EQ-5D or SF-6D. Two studies estimated QALYs based on regression models with DFD or clinical measures as independent variables [38, 42]. According to Jonkers et al. [69] utility scales should be preferred for cost-utility analyses to estimate health effects of quality improvements for depression, compared with QALYs derived from DFD. Moreover, a direct comparison between those QALYs should be avoided [69].

It cannot be ruled out that cost-effectiveness analyses conducted alongside randomized controlled trials of the effect of collaborative care for the treatment of depressive disorders in primary care remain unpublished [70]. Therefore, reporting bias may occur. Yet, it is beyond the scope of this study to examine the retention of cost-effectiveness data to the public. However, there appears to be a strong relationship between the strength and direction of effectiveness results and the presence of a concurrent economic evaluation [70], leading to a potential overestimation of cost-effectiveness of collaborative care compared with usual care. In order to prevent reporting bias, cost-effectiveness analyses should be guided by priorly published study protocols [71].

Generalizability and comparability of the studies included in this review is debatable due to methodological differences and heterogeneous general characteristics. Among others, the study perspectives, settings and effect measures used varied significantly between the studies. For instance, the differences in study perspectives may have led to differences in identification and measurement of costs across studies, since, from a health care perspective, a more restricted selection of cost elements is likely. The settings of the studies were mainly primary care clinics or practices, yet in four different countries. Health care system characteristics across countries of studies included in this review are expected to differ markedly. In addition, nearly half of all studies only included female or elderly patients and patients with co-morbidities, respectively, which are also factors potentially affecting generalizability [72]. In order to improve generalizability, PPP were used in this review to adjust for price level differences across countries [72, 73]. This approach clearly improved comparability across studies. However, it is still a gross adjustment and not a reflection of differences in health care system, unit prices or care provider characteristics between countries [72, 74].

### Limitations of this study

This study has several limitations. First, not more than twelve of 19 studies reported either or both significant differences in costs and effects between the collaborative care groups and usual care groups. Second, the cost-effectiveness of collaborative care compared with usual care was potentially overestimated due to publication bias. Third, the heterogeneity of interventions may have influenced the variation of ICERs as complexity and diversity of collaborative care elements varied across studies. Fourth, the variation of cost categories included in the analyses was considerable. Fifth, the majority of the studies were conducted in the USA. Generalizability to health care systems outside the USA may be limited, because cost-effectiveness of collaborative care may vary across populations and health insurance systems. Last, the review was limited to published studies in English or German, thus potentially introducing bias in the selection of publications.

## Conclusion

Despite our review improved the comparability of study results, cost-effectiveness of collaborative care compared with usual care for the treatment of patients with depressive disorders in primary care is ambiguous depending on willingness to pay for an additional QALY or DFD, respectively. There remains considerable uncertainty due to inconsistent results among included studies. Reviewed cost-effectiveness analyses differed considerably in terms of economic quality, and risk of bias remained uncertain in the majority of studies, due to insufficient reporting. Future cost-effectiveness analyses using QALYs as summary measures and a time horizon of at least one year are needed in order to improve decision-making. Such studies should be conducted in large and representative patient samples from a societal perspective, taking into account indirect costs.

## Supporting Information

S1 PRISMA Checklist PRISMA Checklist [75]. (DOC)

S1 Fig Risk of bias summary: review authors' judgments about each risk of bias item for each included study. +: low risk of bias. −: high risk of bias.?: unclear of risk of bias. Other bias: randomization imbalances (Hay 2012; Schoenbaum 2001; Simon, Katon 2001; Simon 2007), underpowered analysis (Bosmans 2014; Katon 2005; Von Korff 1998), high proportion of missing cost-data (Donohue 2014; Katon 2004), crossing-over (Goorden 2013).
(TIFF)

S2 Fig Risk of bias graph: review authors' judgments about each risk of bias item presented as percentages across all included studies. (TIFF)

S1 Table Methodological quality of studies’ economic evaluations. CHEC-list items: Study population, competing alternatives, research question, economic study design, time horizon, perspective, identification of costs, cost measurement cost valuation, outcome identification, outcome measurement, incremental analysis, discounting, sensitivity analysis, conclusion, generalizability, conflict of interest, ethical issues.
(DOCX)

S2 Table Direct cost elements and mean costs. IG = Intervention Group, CLP = Chilean Pesos, ^§^Analysis was based on two RCT, *Total healthcare costs, **Total societal costs, ^&^significant with p<0.05, ^†^significant with p<0.001.
(DOCX)

S3 Table Intervention elements and mean costs. CLP = Chilean Pesos, ^§^Analysis was based on two RCT.
(DOCX)
