# Supplementary figures and images for: Cost-Effectiveness of Collaborative Care for the Treatment of Depressive Disorders in Primary Care: A Systematic Review
Source: PLoS One. 2015 May 19;10(5):e0123078. doi: 10.1371/journal.pone.0123078 (PMC4437997; doi:10.1371/journal.pone.0123078)

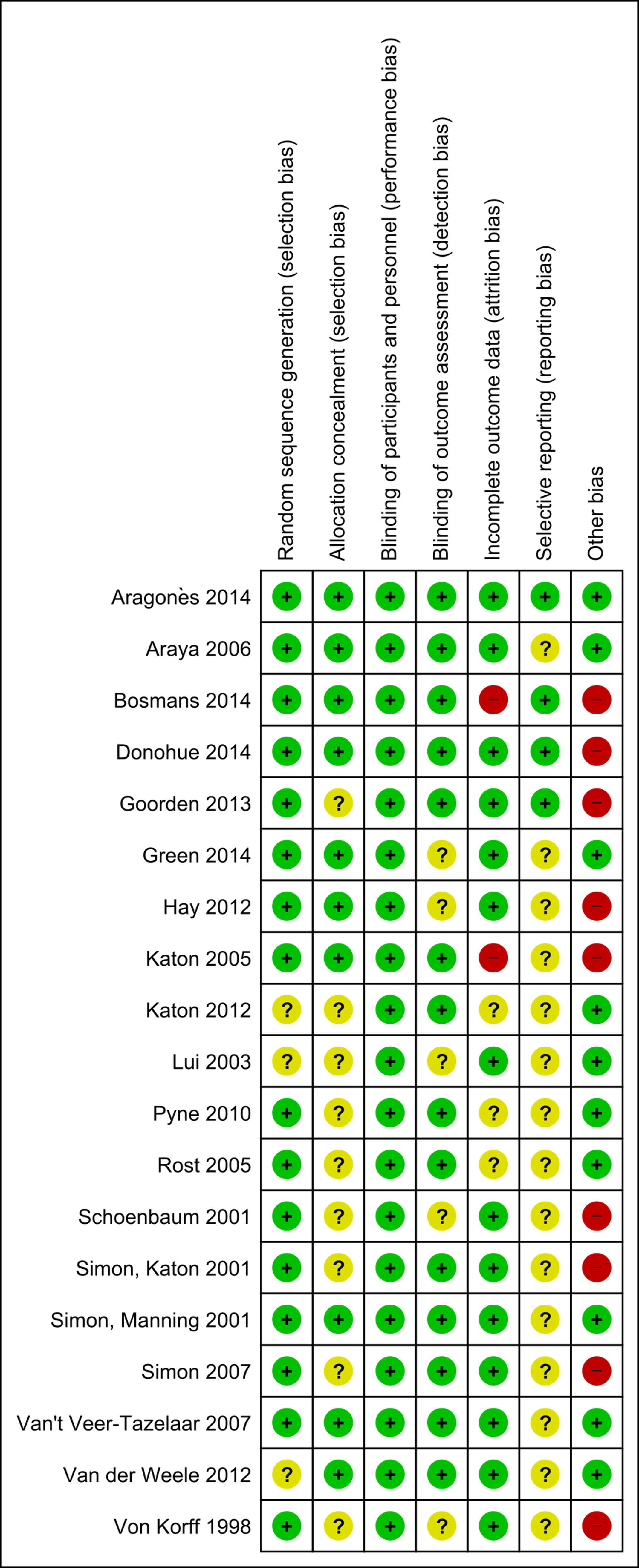

Supplement: S1 Fig — +: low risk of bias. −: high risk of bias.?: unclear of risk of bias. Other bias: randomization imbalances (Hay 2012; Schoenbaum 2001; Simon, Katon 2001; Simon 2007), underpowered analysis (Bosmans 2014; Katon 2005; Von Korff 1998), high proportion of missing cost-data (Donohue 2014; Katon 2004), crossing-over (Goorden 2013). (TIFF) [file pone.0123078.s002.tiff]

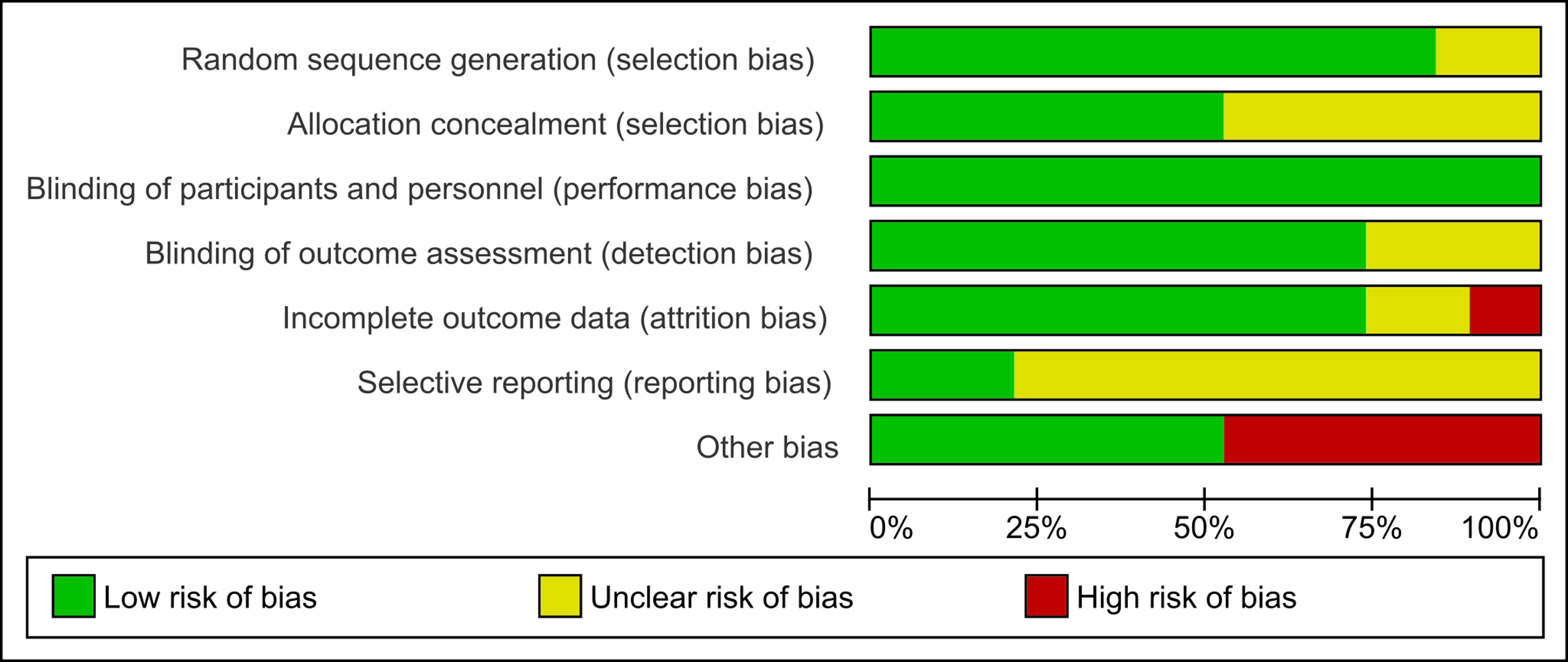

Supplement: S2 Fig — (TIFF) [file pone.0123078.s003.tiff]
