# Supplementary material for: Cost-Effectiveness of Collaborative Care for the Treatment of Depressive Disorders in Primary Care: A Systematic Review
Source: PLoS One. 2015 May 19;10(5):e0123078. doi: 10.1371/journal.pone.0123078 (PMC4437997; doi:10.1371/journal.pone.0123078)
Supplement: S1 Table — CHEC-list items: Study population, competing alternatives, research question, economic study design, time horizon, perspective, identification of costs, cost measurement cost valuation, outcome identification, outcome measurement, incremental analysis, discounting, sensitivity analysis, conclusion, generalizability, conflict of interest, ethical issues. (DOCX) [file pone.0123078.s004.docx]

S1 Table. Methodological quality of studies’ economic evaluations

| **Study** | **Score on the C19-item CHEC-list for economic evaluations [28]** |
| --- | --- |
| **Aragonès et al. 2014 [33, 76, 77]** | 16 |
| **Araya et al. 2006 [34]** | 11 |
| **Bosmans et al. 2014 [35]** | 17 |
| **Donohue et al. 2014 [51, 78, 79]** | 12 |
| **Goorden et al. 2013 [36, 80]** | 16 |
| **Green et al. 2014 [50, 81, 82]** | 13 |
| **Hay et al. 2012 [20, 37]** | 15 |
| **Katon et al. 2012 [38, 83]** | 14 |
| **Katon et al. 2005 [39, 84, 85]** | 11 |
| **Liu et al. 2003 [40]** | 12 |
| **Pyne et al. 2010 [21, 22, 41]** | 12 |
| **Rost et al. 2005 [42, 86, 87]** | 12 |
| **Schoenbaum et al. 2001 [43, 88, 89]** | 15 |
| **Simon et al. 2007 [44, 90, 91]** | 14 |
| **Simon, Katon et al. 2001 [45, 92]** | 8 |
| **Simon, Manning et al. 2001 [46]** | 13 |
| **Van der Weele et al. 2012 [47]** | 16 |
| **van't Veer-Tazelaar et al. 2010 [48, 93]** | 15 |
| **Von Korff et al. 1998 [13, 49, 52]** | 8 |

CHEC-list items: Study population, competing alternatives, research question, economic study design, time horizon, perspective, identification of costs, cost measurement cost valuation, outcome identification, outcome measurement, incremental analysis, discounting, sensitivity analysis, conclusion, generalizability, conflict of interest, ethical issues
