# Supplementary material for: Cost-Effectiveness of Collaborative Care for the Treatment of Depressive Disorders in Primary Care: A Systematic Review
Source: PLoS One. 2015 May 19;10(5):e0123078. doi: 10.1371/journal.pone.0123078 (PMC4437997; doi:10.1371/journal.pone.0123078)
Supplement: S2 Table — IG = Intervention Group, CLP = Chilean Pesos, §Analysis was based on two RCT, *Total healthcare costs, **Total societal costs, &significant with p<0.05, †significant with p<0.001. (DOCX) [file pone.0123078.s005.docx]

S2 Table. Direct cost elements and mean costs

| **Article** | **Follow up in months** | **Direct medical and non-medical elements** | **Mean cost IG (95% CI)** | **Mean costs IG in US$PPP (95% CI)** | **Incremental overall mean costs in US$PPP** |
| --- | --- | --- | --- | --- | --- |
| **Aragonès et al. 2014 [33]** | 12 | Medication (antidepressants, other psychotic drugs), outpatient care (primary care, outpatient specialized care), inpatient care (hospital) | € 776 (SD 664) | 1,110 (SD 950) | 261 |
| **Araya et al. 2006 [34]** | 6 | Medication (antidepressants, benzodiazepine), outpatient care (primary care, psychiatrist) | CLP 25,362 (22,791 to 29,854) | 110 (99 to 130) | 46 |
| **Bosmans et al. 2014 [35]** | 10 | Medication, outpatient care (primary care, other), inpatient care (clinic, hospital) | € 4,284 (SD 595) | 5,537 (SD 769) | 1,083 |
| **Donohue et al. 2014 [51]** | 12 | Outpatient care (primary care, specialty care, laboratory, imaging, emergency care, other), inpatient care (medical, surgical) | US$ 18,173 (SD 8,316) | 21,399 (SD 9,792) | −529* |
| **Goorden et al. 2013 [36]** | 12 | Medication, outpatient care (primary care, psychologist, psychiatrist, occupational medicine), other medical (specialist, mental health care institute, paramedic, alternative medicine), non-medical (social worker, self-help group, daycare) | € 3,900 | 5,036 | −904 |
| **Green et al. 2014 [50]** | 12 | Outpatient care (primary care, community care), inpatient care (hospital, day hospital), emergency care, non-medical care (day services and care) | £ 1,887 (SD 3,714) | 2,725 (SD 5,365) | 457 |
| **Hay et al. 2012 [37]** | 18 | Medication, outpatient care, inpatient care, laboratory, emergency, other | *Not given* | *Not given* | - |
| **Katon et al. 2012 [38]** | 24 | Medication, outpatient care (primary care, specialty care, mental health, ambulatory surgery, other), emergency, laboratory and radiology, | US$ 21,607 (17,723 to 25,493) | 22,680 (18,603 to 26,759) | 1,385 |
| **Katon et al. 2005 [39]** | 24 | Medication (antidepressants, other), outpatient care (mental health, other), inpatient care (mental health, medical) | US$ 16,175 (14,522 to 17,829) | 18,456 (16,570 to 20,343) | 373 |
| **Liu et al. 2003 [40]** | 9 | Antidepressant medication, outpatient care (primary care, mental health) | US$ 7,946 (5,582 to 103,310) | 10,187 (7,156 to 132,446) | 1,483 |
| **Pyne al. 2010 [41]** | 12 | Medication (outpatient), outpatient care, inpatient care | € 7,730 (SD 1,485) | 10,580 (SD 2,033) | 3,761 |
| **Rost et al. 2005 [42]** | 24 | Medication (outpatient), outpatient care (mental health, emergency department) | *Not given* | *Not given* | - |
| **Schoenbaum et al. 2001 [43]** | 24 | *None specified* | US$ 3,416 | 4,543 | −557 (QI-−meds) |
|  |  |  | US$ 3,350 | 4,455 | −645 (QI-therapy) |
|  |  |  | US$ 3,381 | 4,496 | −604 (pooled) |
| **Simon et al. 2007 [44]** | 24 | Medication (antidepressants, other), outpatient care (mental health, medical, other), emergency care, diagnostic services, screening, other outpatient services | US$ 15,100 | 16,285 | −982 |
| **Simon, Katon et al. 2001 [45]** | 6 | Medication (antidepressants, other), outpatient care (mental health, other), inpatient care (mental health, other) | US$ 2,406 (1,769 to 3,218) | 3,013 (2,216 to 4,030) | 371 |
| **Simon, Manning et al. 2001 [46]** | 12 | Medication, outpatient care (medical, clinic, other), inpatient care | US$ 5,549 (4,313 to 6,784) | 6,950 (5,402 to 8,497) | 2,054 |
| **Van der Weele et al. 2012 [47]** | 12 | Medication, outpatient care (primary, care, psychiatrist, psychologist, other), inpatient care (psychiatry, hospital, daycare), paramedical care (physiotherapist, other), non-medical (care home, nursing home, nursing home care, formal home care, informal home care) | € 14,024 | 21,001 | 6,996** (age 75-79) |
|  |  |  | € 16,087 | 24,091 | −860** (age ≥80) |
| **van't Veer-Tazelaar et al. 2010 [48]** | 12 | Medication, outpatient care (primary care, psychologist), non-medical costs (home care, meals on wheels, social activities, informal care) | € 2,985 (SD 1,711) | 3,944 (SD 2,261) | 703 |
| **Von Korff et al. 1998 [49]^§^** | 7 | Antidepressant medication, outpatient care (primary care, mental health) | US$ 1,337 | 1,860 | 678 (reference year 1995) |
|  |  |  | US$ 1,182 | 1,615 | 361 (reference year 1996) |

IG = Intervention Group, CLP = Chilean Pesos

^§^Analysis was based on two RCT

*Total healthcare costs

**Total societal costs

^&^significant with p<0.05

^†^significant with p<0.001
