# Supplementary material for: Cost-Effectiveness of Collaborative Care for the Treatment of Depressive Disorders in Primary Care: A Systematic Review
Source: PLoS One. 2015 May 19;10(5):e0123078. doi: 10.1371/journal.pone.0123078 (PMC4437997; doi:10.1371/journal.pone.0123078)
Supplement: S3 Table — CLP = Chilean Pesos, §Analysis was based on two RCT. (DOCX) [file pone.0123078.s006.docx]

S3 Table. Intervention elements and mean costs

| **Article** | **Follow up in months** | **Intervention elements** | **Mean cost (95% CI)** | **Mean costs in US$PPP (95% CI)** |
| --- | --- | --- | --- | --- |
| **Aragonès et al. 2014 [33]** | 12 | Care manager | € 71 | 102 |
| **Araya et al. 2006 [34]** | 6 | Group psychoeducational training, patient support, group psychoeducation liaison | CLP 5,942 | 26 |
| **Bosmans et al. 2014 [35]** | 10 | Screening, watchful waiting, activity scheduling, life review | € 209 (SD 21) | 270 (SD 27) |
| **Donohue et al. 2014 [51]** | 12 | Nurse care manager telephone contacts, documentation, physician supervision, tracking registry, workbook, mailings, other miscellaneous support | US$ 460 | 542 |
| **Goorden et al. 2013 [36]** | 12 | Occupational physician care manager | € 208 (SD 246) | 269 (SD 318) |
| **Green et al. 2014 [50]** | 12 | Care manager, clinical supervision | £ 273 | 394 |
| **Hay et al. 2012 [37]** | 18 | Diabetes depression clinical specialists, patient navigator services | US$ 515 (469 to 561) | 541 (492 to 589) |
| **Katon et al. 2012 [38]** | 24 | Supervision by physicians, record keeping, outreach efforts, information system support | US$ 1,204 (1,092 to 1,514) | 1,264 (1,146 to 1,589) |
| **Katon et al. 2005 [39]** | 24 | Patient contacts, depression care manager, team psychiatrists, primary care experts, intervention materials | US$ 591 (574 to 608) | 674 (655 to 694) |
| **Liu et al. 2003 [40]** | 9 | Social work follow-up calls, team treatment meetings | US$ 182 (168 to 196) | 233 (215 to 251) |
| **Pyne al. 2010 [41]** | 12 | Fixed intervention costs, time spent by intervention personnel delivering the intervention | € 794 | 1,087 |
| **Rost et al. 2005 [42]** | 24 | Screening, care manager, record keeping, physician, overhead | US$ 321 | 412 |
| **Schoenbaum et al. 2001 [43]** | 24 | Screening, intervention materials, initial nurse specialist assessments, supervision of nurses and therapists | *Not given* | *Not given* |
| **Simon et al. 2007 [44]** | 24 | Intervention services | US$ 545 (SD 222) | 588 (SD 239) |
| **Simon, Katon et al. 2001 [45]** | 6 | Consultation with collaborative care psychiatrist | US$ 184 (168 to 201) | 230 (210 to 252) |
| **Simon, Manning et al. 2001 [46]** | 12 | Screening, treatment coordinator | US$ 135 (132 to 138) | 169 (165 to 173) |
| **Van der Weele et al. 2012 [47]** | 12 | Individual consultations, course sessions | € 311 | 466 (age 75-79) |
|  |  |  | € 251 | 376 (age ≥80) |
| **van't Veer-Tazelaar et al. 2010 [48]** | 12 | Watchful waiting, bibliotherapy, problem-solving treatment, screening | € 563 (SD 361) | 744 (SD 477) |
| **Von Korff et al. 1998 [49]^§^** | 7 | Intervention visits | US$ 418 | 582 (reference year 1995) |
|  | 7 | Intervention visits, psychiatric supervision | US$ 434 | 593 (reference year 1996) |

CLP = Chilean Pesos

^§^Analysis was based on two RCT
